# Preparation and Application of Carboxylated Graphene Oxide Sponge in Dye Removal

**DOI:** 10.3390/ijerph14111301

**Published:** 2017-10-26

**Authors:** Lianqin Zhao, Sheng-Tao Yang, Shicheng Feng, Qiang Ma, Xiaoling Peng, Deyi Wu

**Affiliations:** 1 School of Environmental Science and Engineering, Shanghai Jiao Tong University, No. 800 Dongchuan Road, Shanghai 200240, China; zlqqlove@sjtu.edu.cn; 2 College of Chemistry and Environment Protection Engineering, Southwest University for Nationalities, Chengdu 610041, China; 18328594054@163.com (S.F.); qiang9322@outlook.com (Q.M.); 18328092638@163.com (X.P.)

**Keywords:** graphene oxide, carboxylation, adsorption, methylene blue, water treatment

## Abstract

Spongy graphene is a newly developed adsorbent of high performance for water treatment. Proper functionalization is an efficient approach to improve the adsorption capacity of graphene adsorbents. In this study, we prepared graphene oxide (GO), functionalized it with carboxyl groups to produce carboxylated GO (GO-COOH) dispersion, and lyophilized the GO-COOH dispersion to obtain the GO-COOH sponge. The adsorption isotherm, kinetics, thermodynamics, influencing factors, and regeneration of the adsorption of dye methylene blue (MB) on GO-COOH sponge were evaluated in batch experiments. The adsorption capacity of GO-COOH sponge was measured as 780 mg/g, which was nearly twice that of GO sponge (446 mg/g). The adsorption isotherm could be well described by the Freundlich model with a *K_F_* of 508 (L/mg)^1/n^. The adsorption kinetic was nicely fitted by pseudo-first-order model with a *k*_1_ of 0.00157·min^−1^. In thermodynamics analysis, the negative Δ*G* indicated the spontaneous nature of adsorption on GO-COOH sponge. The adsorption process was endothermic and was driven by the increase of entropy. Higher pH benefited the removal of MB by GO-COOH sponge and the ionic strength had no meaningful effect. The regeneration was poor due to the strong electrostatic interaction between MB and the GO-COOH sponge. The results collectively suggested that carboxylation increased the adsorption performance of GO sponge.

## 1. Introduction

Dye pollution is one of the most serious environmental pollutions that is usually released from textile, printing, and pharmaceutical factories [1,2,3]. Many dye pollutants are organic molecules containing polar functional groups, which are soluble in water and could easily diffuse into the environment. Once spreading into the environment, the dye pollutants are polychrome and visible, which is a great threat to people and the environment [4,5]. Therefore, there is a great need to remediate the dye pollutants before their release into the water system [6,7,8]. There are many techniques for this purpose, such as the active sludge method, Fenton reaction, electrolysis, and adsorption [9,10]. In particular, adsorption technology holds several advantages, such as easy operation, fast decoloration, and excellent chemical oxygen demand (COD) removal efficiency [11,12]. The major limit of the adsorption technology is the low adsorption capacity of traditional adsorbents. Therefore, the exploration of effective and environmentally friendly adsorbents is in highly demand and has become the main direction of adsorption studies.

Recently, the nanoadsorbents are applied in wastewater remediation due to their large surface area and the controllable surface chemistry of nanomaterials [13,14,15,16]. Graphene is composed of six-member-ring sp^2^ carbon atoms that form single layers. All the carbon atoms of graphene are surficial atoms and both surfaces of a graphene layer are available to the pollutants. In addition, the oxidation degree of graphene can be well controlled to produce abundant oxygen containing groups. Therefore, graphene adsorbents are regarded as the most promising nanoadsorbents for water treatment. The first demonstration was reported by Yang et al. in 2010 [17]. Graphene oxide (GO) has been shown to adsorb Cu^2+^ strongly. A similar attempt was performed on cationic dye methylene blue (MB), and the adsorption of MB on GO dispersion was mainly driven by electrostatic interaction [18]. Other groups have also used GO and its derivatives to adsorb diverse pollutants, such as heavy metal ions, dyes, antibiotics, pesticides, and oils [19]. Further investigation indicated that the oxygen concentrations of graphene regulated the adsorption performance in treating MB solutions [20,21,22]. Higher oxygen group abundance led to larger adsorption capacities because of the stronger electrostatic interaction. Beyond the oxidation degree of GO, more recent studies concerned the separation of GO from water after treatment, and the results suggested that spongy GO and other spongy graphene adsorbents were better for practical use. To improve the adsorption capacity of graphene sponge for cationic pollutants, there are two possible approaches, namely increasing the oxygen content and introducing other groups that have stronger affinities to the pollutants. GO already has a very high oxygen content, which hinders the further increase of oxygen content. Thus, a better choice is to introduce groups of higher affinity to the pollutants. Following this strategy, we previously reduced and doped GO with cysteine, and found that the resulting S-doped graphene possessed high binding strength with heavy metal ions [23]. However, the doped sulfur only interacts strongly with metal ions. For cationic dyes, the involvement of other stronger groups should be explored.

Many studies have shown that the hydroxyl groups and the ether bonds of GO could be converted into carboxyl groups easily [24,25]. Carboxyl groups deprotonate stronger than hydroxyl groups and ether bonds, and thus have higher binding strength with respect to cationic molecules. GO-COOH dispersion could be applied in the removal of ionic dyes [26]. Sun et al. [27] investigated the removal of U^6+^ by GO-COOH experimentally and theoretically with a maximum adsorption capacity of 103.09 mg/g. Based on these findings, we speculated that the strategy of converting hydroxyl groups and the ether bonds into carboxyl groups could improve the performance of GO sponge as well. To verify our hypothesis, we prepared carboxylated GO (GO-COOH) sponge through an electrophilic addition reaction, investigated its adsorption performance in treating MB solution, and compared it with GO sponge. The GO-COOH sponge was characterized by infrared spectroscopy (IR), Raman spectroscopy, thermogravimetric analysis (TGA), X-ray photoelectron spectroscopy (XPS), scanning electron microscope (SEM), and transmission electron microscope (TEM). The adsorption isotherm of MB on GO-COOH sponge was measured and fitted to the Freuendlich model, the Langmuir model, and the Temkin model. The adsorption kinetics and thermodynamics were analyzed to reveal the adsorption behaviors of MB on GO-COOH sponge. The influences of pH and ionic strength on the adsorption were evaluated as well. The main novelty of our study was the introduction of additional carboxyl groups onto graphene sponge to improve its adsorption performance, which provided a new strategy of enhancing the spongy graphene adsorbents. The implication to the application of GO-COOH sponge in water treatment is discussed.

## 2. Materials and Methods

### 2.1. Materials

Graphite was obtained from Sinopharm Chemical Reagent Co., Ltd., Shanghai, China. MB was bought from Solarbio Biochemical Reagent Co., Beijing, China. NaOH was purchased from Ruijinte Chemical Reagent Co., Ltd., Tianjin, China. ClCH_2_COOH was obtained from Damao Chemical Reagent Co., Ltd., Tianjin, China. The rest were all of analytical grade.

### 2.2. Synthesis of GO-COOH Sponge

GO was prepared following the modified Hummers methods [28]. GO suspension was sonicated for 1 h to disperse homogeneously. The homogeneous GO dispersion was lyophilized to produce GO sponge. The GO sponge (0.2 g) was dissolved in purified water under ultrasonic treatment for 3 h followed by the addition of NaOH (12 g) and ClCH_2_COOH (10 g). The mixture was then sonicated for another 3 h to obtain GO-COOH. The product was treated with thermal filtration, dialysis against water (48 h), and lyophilization to obtain the final product GO-COOH sponge [29].

The as-prepared GO sponge and GO-COOH sponge were characterized by IR (Magna-IR 750, Nicolet, SpectraLab Scientific Inc., Alexandria, VA, USA), Raman (inVia, Renishaw, UK), TGA (Q500, TA Instruments, New Castle, DE, USA), XPS (Kratos, London, UK), SEM (Quanta 200FEG, FEI, Eindhoven, The Netherlands), and TEM (JEM-200CX, JEOL, Tokyo, Japan).

### 2.3. Adsorption Isotherm

The adsorption of MB on GO-COOH sponge was evaluated by batch experiments. Each 5.0 mg of GO-COOH sponge was mixed with MB (8.0 mL, 50–1000 mg/L, pH 7.3) in a plastic tube. The tubes were shaken on a thermostat at 303 K (CHA-S, Jintan Hankang Electronic Co., Jintan, China) at 100 rpm for 24 h to reach the equilibrium. Then, the mixtures were centrifuged at 12,000 rpm (TG16 W, Pingfan Instrument and Meter Co., Changsha, China) to obtain the supernatants for the absorbance measurements on a spectrometer (UV-1800, Shanghai Mapada Instrument Co., Ltd., Shangai, China). All the experimental data were presented as mean ± standard deviation (mean ± SD). The equilibrium concentration (*C_e_*) was obtained referring to the standard curve of MB (*Abs* = 0.2197*Ce*, *R* = 0.9994), and the equilibrium adsorption capacity (*q_e_*) could be calculated by (*C*_0_ − *C_e_*)/*C_sponge_*. The *q_e_* values of GO-COOH sponge were subjected to different isothermal models following previous reports, including the Langmuir model, the Freundlich model, and the Temkin model.

### 2.4. Kinetics and Thermodynamics

To evaluate the adsorption kinetics, the GO-COOH sponge (5.0 mg) was mixed with 8.0 mL of MB (900 mg/L, pH 7.3), shaken on a thermostat at 303 K for different intervals (5–1020 min), and centrifuged at 12,000 rpm for 5 min. The MB concentration was symbolized as *C_t_*, and the adsorption capacity *q_t_* could be calculated by (*C*_0_ − *C_e_*)/*C_sponge_*. The data was analyzed by the pseudo-first-order model, the pseudo-second-order model, and the intraparticle diffusion model.

To investigate the adsorption thermodynamics, the mixtures of GO-COOH sponge (5.0 mg) and 8.0 mL of MB (900 mg/L, pH 7.3) were incubated at different temperatures (303 K, 313 K, 323 K and 333 K) for 24 h. The adsorption data were fitted to Equation (1) to calculate the thermodynamics parameters [30]. The distribution coefficient *K_d_* was calculated by *q_e_/c_e_*. The Δ*G* was calculated accordingly.

(1) $$\ln k_{d}=-\frac{\Delta H}{RT}+\frac{\Delta S}{R}$$

### 2.5. Influence of pH and Ionic Strength

To investigate the influence of pH, the initial pH values of the MB solutions were adjusted to the range of 3–11 by an HCl or NaOH solution. At each pH condition, the GO-COOH sponge (5.0 mg) was mixed with 8.0 mL of MB (900 mg/L) for the determination of *q_e_* following the aforementioned protocol. The pH was adjusted only with HCl and NaOH rather than using a buffer solution because, during the adsorption, H^+^ would be released due to the H^+^ exchange between carboxyl groups and MB molecules. Using a simple acid and base could reflect the real situation during water treatment. Similarly, the evaluation of ionic strength was taken by mixing the GO-COOH sponge with an MB solution containing NaCl. The GO-COOH sponge (5.0 mg) was mixed with 8.0 mL of MB (900 mg/L, Na^+^ concentration: 0–100 mM). The *q_e_* values were obtained as described above.

### 2.6. Regeneration

To evaluate the recycling of GO-COOH sponge, the residue was washed with acidic water (pH 3). The adsorption capacity of recycled GO-COOH sponge was measured as aforementioned up to the recycle number of 5. The recycled GO-COOH sponge was analyzed by IR and SEM to reveal the potential changes.

## 3. Results and Discussion

### 3.1. Characterization of GO-COOH Sponge

**Figure 1:**
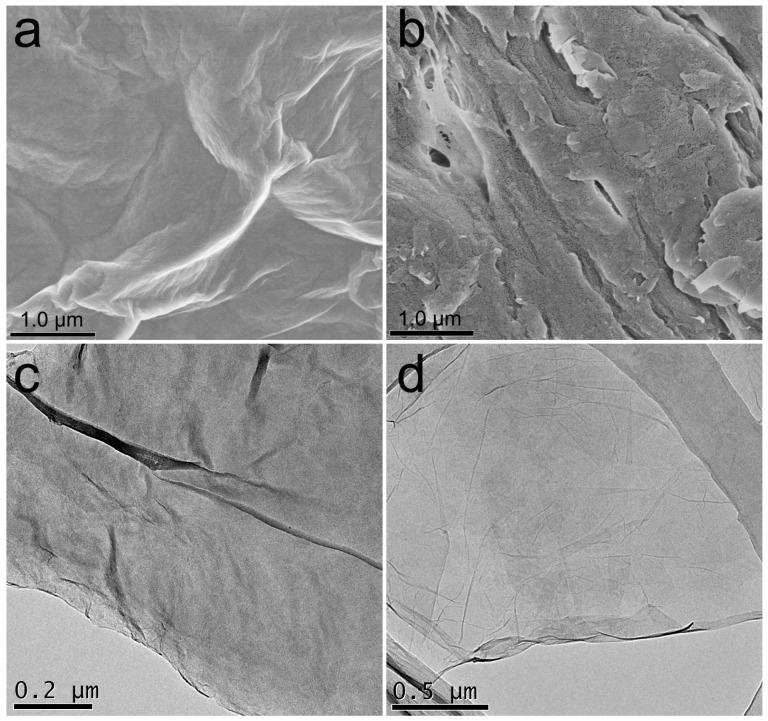
Characterization of graphene oxide (GO) sponge (**a**,**c**) and carboxylated GO (GO-COOH) sponge (**b**,**d**). (**a**,**b**) SEM images; (**c**,**d**) TEM images.

The appearances of GO sponge before and after carboxylation were similar. As illustrated in Figure 1a, the precursor material GO sponge was loosely stacked with some wrinkles under SEM. For GO-COOH sponge, the stacking and wrinkles still presented (Figure 1b), while the sheets of GO-COOH sponge had some pores on the surface. This might be due to the destruction of GO sheets during the carboxylation. The sheets were clearly recognized under TEM for both sponges (Figure 1c,d). For the preparation of TEM specimens, GO and GO-COOH sponges were sonicated in water, so the images only reflect the sheet structure rather than the 3D structure. The main difference was that more single-layer sheets were found in the GO-COOH sponge. It should be noted that without sonication GO-COOH sponge could not be dispersed in water without sonication treatment.

**Figure 2:**
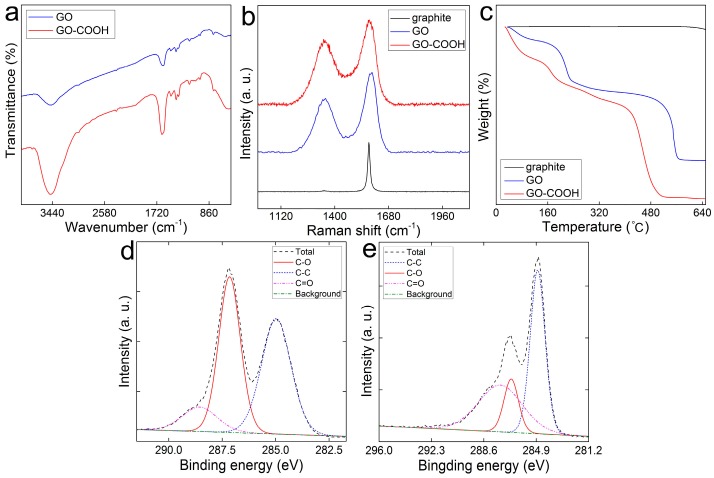
Characterization of graphite, GO sponge and GO-COOH sponge. (**a**) IR spectra; (**b**) Raman spectra; (**c**) TGA data; (D&E) C1s XPS spectra of GO sponge (**d**) and GO-COOH sponge (**e**).

To confirm the successful carboxylation of GO, GO sponge and GO-COOH sponge were compared in several characterizations. First, the successful addition of -COOH to GO was evidenced by the IR spectra. Comparing to the IR spectrum of GO, the remarkable increase near 3470 cm^−1^ was attributed to the increased -COOH groups (Figure 2a). In the Raman spectra, the initial graphite had a good in-plane sp^2^ carbon vibration in 1578 cm^−1^ (G band) with no remarkable defect in its lattice (Figure 2b), because there was no obvious D band (near 1350 cm^−1^). After being converted into GO by the modified Hummers method, there was an evident D band near 1350 cm^−1^ and G band at 1590 cm^−1^. The intensity ratios of D band and G band (*I_D_*/*I_G_*), an important parameter in evaluating the formation of edge-defects and the introduction of high leveled crystalline structural disorder (attributing to the formation of sp^3^-C after functionalization), were 0.674 for GO sponge and 0.763 for GO-COOH sponge. This suggested that GO-COOH sponge possessed more disordered structures than GO sponge. After the carboxylation, the center of the G band in GO-COOH sponge shifted to 1582 cm^−1^, which was lower than that in GO sponge. The Raman shift was attributed to the weakened interlayer forces and the increased stacking of graphene sheet. TGA measurement was also adopted to characterize the thermal stability of GO-COOH sponge. There was a slight weight loss for GO sponge and GO-COOH sponge from 0 to 100 °C due to the evaporation of adhered water molecules on the surface. For GO sponge, there were two steps of weight loss at 192 °C and 529 °C. The first weight decrease was attributed to the conversion of O-containing groups into carbon oxides and H_2_O [31,32]. The second step was attributed to the combustion of GO carbon skeleton [33]. For GO-COOH sponge, there was an apparent weight loss at about 180 °C, which might have resulted from the decomposition of O-containing groups [34]. Following a steady decrease in the range of 200 to 400 °C, there was a sharp decrease of weight loss for about 40% in the range of 410 to 510 °C. The large amount of weight loss could be attributed to the loss of the more stable O-containing groups [35]. Additionally, in the C1s XPS spectrum (Figure 2d), there were several peaks at 284.95 eV (-C-C), 287.15 eV (-C-O) and 288.55 eV (-C=O) in GO sponge, respectively. For GO-COOH sponge (Figure 2e), the intensity at 284.8 eV (-C-C) decreased, and the signals for -C-O and -C=O at 286.65 eV and 287.45 eV increased, indicating the successful introduction of -COOH groups into GO structure [36].

### 3.2. Adsorption of MB on GO-COOH Sponge

**Figure 3:**
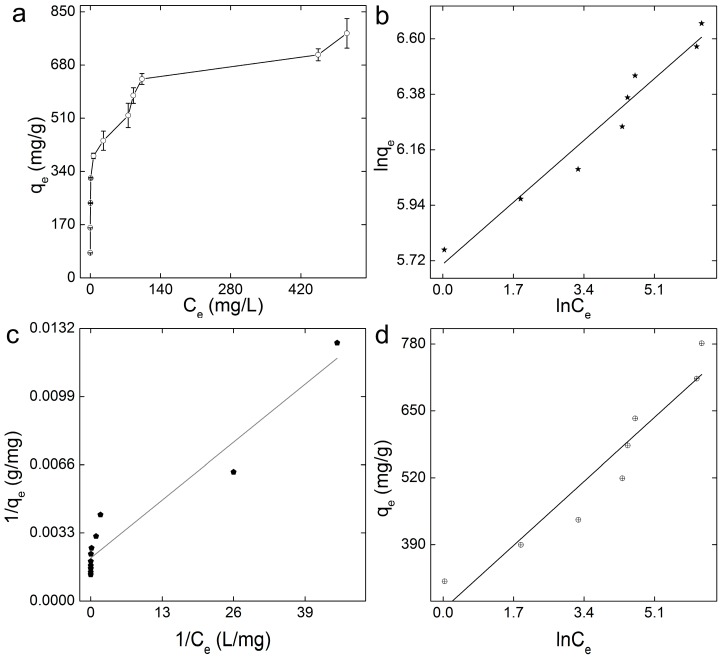
Adsorption of methylene blue (MB) onto GO-COOH sponge. (**a**) Adsorption isotherm. Data represent mean ± SD (*n* = 3); (**b**) the Freundlich model; (**c**) the Langmuir model; (**d**) the Temkin model.

The adsorption performance of GO-COOH sponge for MB was evaluated in batch experiments. MB was used as the model pollutant, because it was positively charged and would have electrostatic interaction with the carboxyl groups on GO-COOH sponge. As shown in Figure 3a, the *q_e_* increased with the growth of *C_e_* and the maximum adsorption capacity (*q_m_*) was obtained. At the *C_e_* of 900 mg/L, the equilibrium adsorption capacity was about 780 mg/g. To exhibit more about the adsorption data, different isothermal adsorption models were applied, including the Langmuir model (*R* = 0.962), the Freundlich model (*R* = 0.979), and the Temkin model (*R* = 0.956). The fitting equations are shown in Table 1. According to the *R* values, the Freundlich model had a better description of the MB adsorption on GO-COOH sponge (Figure 3b), and the adsorption was a heterogeneous process. The equilibrium constant (*K_F_*) of the model was about 508 (L/mg)^1/n^, reflecting the large adsorption capacity. Another equilibrium constant (*n*) was a good indicator of the tendency of adsorption [37]. In our experiment, the calculated *n* was about 6.944, so the adsorption process tended to be successful.

Comparing to the adsorption capacity of GO sponge, the carboxylation increased the adsorption capacity significantly from 446 mg/g for GO sponge to 780 mg/g for GO-COOH sponge. Moreover, the adsorption performance of GO-COOH sponge was also larger than that of GO dispersion (714 mg/g) [18]. The increased adsorption performance of GO-COOH sponge should be due to the stronger binding strength between graphene sheets and MB molecules. The adsorption capacity of GO-COOH had already ranked it among the most effective graphene sponges for dye removal. The adsorption performance of graphene materials for MB is listed in Table 2.

### 3.3. Adsorption Kinetics and Thermodynamics

**Figure 4:**
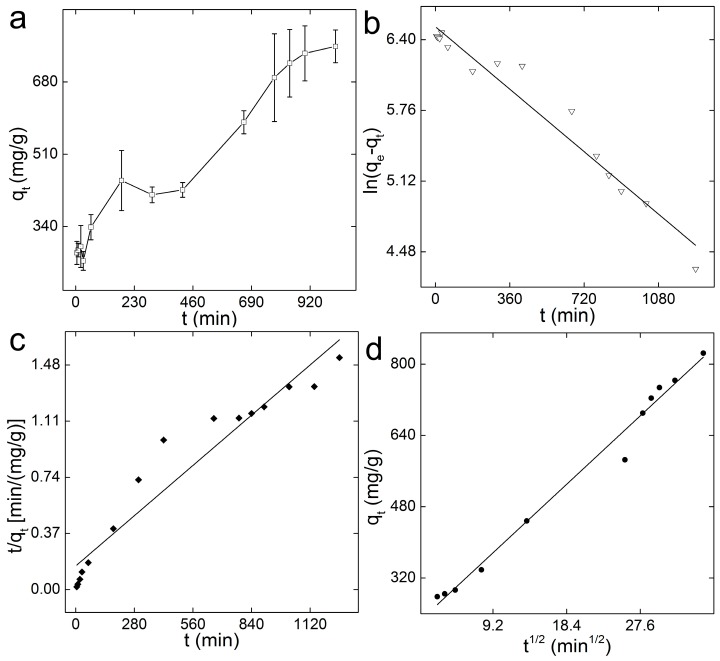
Kinetics analysis of the adsorption of MB onto GO-COOH. (**a**) Adsorption as a function of time. Data represent mean ± SD (*n* = 3); (**b**) the pseudo-first-order model; (**c**) the pseudo-second-order model; (**d**) the intra-particle model.

In the kinetics analyses, the adsorption capacity of GO-COOH sponge for MB (*q_t_*) increased gradually as time elapsed (Figure 4a). When time increased to 900 min, the adsorption capacity reached a plateau and kept nearly constant. The equilibrium time of 15 h was a bit long for practical applications. The long equilibrium time should be due to the spongy structure of GO-COOH sponge, where the diffusion of MB to the inner pores required much more time. For practical uses, a possible approach could be to use less GO-COOH sponge. The adsorption process of MB on GO-COOH sponge could be well described by pseudo-first-order model (*R* = 0.98) with a *k*_1_ value of 0.00157 min^−1^, which was smaller than that of GO sponge for MB removal (*k*_1_ value was 0.0053 min^−1^). The calculated *q_e_* was about 679 mg/g, lower than the experimental *q_e_* (780 mg/g). For the pseudo-second-order model, the *R* value was 0.96 and the calculated *q_e_* was 847 mg/g. The corresponding *k*_2_ was 8.95 × 10^−6^ mg/(g·min). The data could be fitted to the intraparticle model for the mechanism exploration (Figure 4d). The whole removal process could be ascribed into one removal step. The *C* value of 222.9 mg/g, reflecting a large boundary effect. The large boundary effect inhibited the facile diffusion of MB onto the reactive adsorbent surface, so the achievement of the adsorption equilibrium needed more time. Meanwhile, the fitted line did not pass through the origin point, reflecting that the adsorption of MB onto GO-COOH sponge included two types of diffusion, namely intraparticle diffusion and surface diffusion.

**Figure 5:**
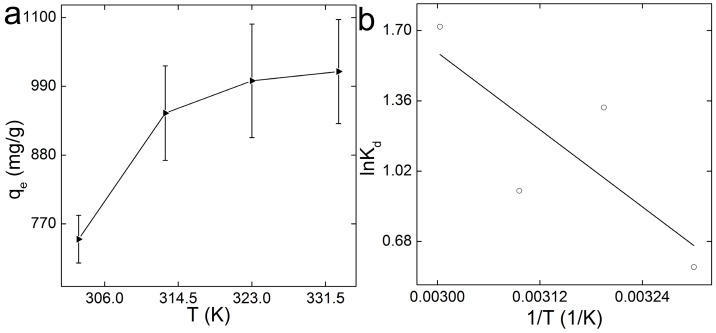
Thermodynamics analyses of the adsorption of MB on GO-COOH sponge. (**a**) Adsorption as a function of contact temperature. Data represent mean ± SD (*n* = 3). (**b**) Thermodynamic analysis.

The thermodynamics parameters have profound implications in evaluating the adsorbent. As illustrated in Figure 5a, there was a slight increase of adsorption capacity along with the increase of temperature. The thermodynamics parameters were calculated by fitting the data to Equation (1) (Figure 5b), and the obtained values are listed in Table 3. In the investigated temperature range, the Δ*G* values were all negative, and the values decreased with the increase of temperature. This suggests that the adsorption of MB on GO-COOH sponge was spontaneous and more favorable at higher temperatures. The facile and spontaneous adsorption process was consistent with the large coefficient *n* in Freundlich model analysis. The Δ*H* value was 25.85 kJ/mol, indicating that the adsorption process was endothermic. The Δ*S* was about 90.81 J/(mol·K), indicating that the interaction of MB with GO-COOH sponge increased the randomness at the contact surface of the solid and the solution. Combined with the negative Δ*G*, we concluded that the removal of MB by GO-COOH sponge could be mainly attributed to the entropy effect.

### 3.4. Influence of pH and Ionic Strength

**Figure 6:**
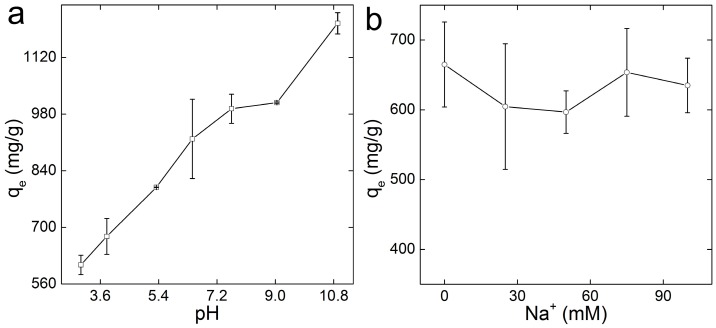
Adsorption of MB on GO-COOH sponge at different pH (**a**) and ionic strength (**b**) values.

A good adsorbent should be able to bear severe environmental conditions. The most frequently concerned parameters are the pH value and ionic strength of the polluted water [45]. The pH value determined the deprotonation of -COOH and -OH groups, which was vital for the adsorption of positively charged pollutants. The adsorption capacities of GO-COOH for MB increased dramatically with the growth of pH in the range of 3–11 (Figure 6a). The increased adsorption capacity could be attributed to the stimulated deprotonation of -COOH groups and hence enhanced the interaction between GO-COOH sponge and MB molecule. Comparing with GO sponge, whose capacity increased only 3% from pH 2–12, the influence of pH was more obvious for GO-COOH. This was reasonable that -COOH deprotonated much easier than -OH groups. Another issue that should be noted is that the strong acidic or strong alkaline conditions were not preferred for practical use. It was not economically or environmentally friendly to use GO-COOH sponge at pH 11, although the adsorption capacity was higher. Unlike pH, the ionic strength had no evident effect on the removal of MB by GO-COOH sponge (Figure 6b). The adsorption capacity of GO-COOH sponge kept nearly constant in the ionic strength range from 0 to 100 mM. The results implied that MB bonded stronger to GO-COOH sponge than Na^+^.

### 3.5. Recycling of GO-COOH

**Figure 7:**
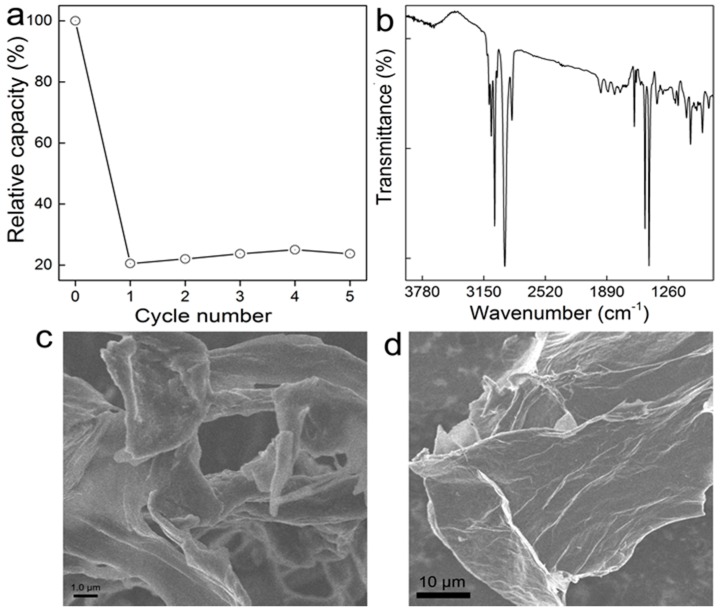
Recycling of MB adsorbed GO-COOH by lyophilization and oven dry treatments. (**a**) Regeneration percentage. Data represent mean ± SD (*n* = 3). (**b**) IR of GO-COOH after regeneration. (**c**,**d**) SEM images of GO-COOH sponge after regeneration.

Practically, a good adsorbent should possess advantages of an excellent removal performance towards pollutants and an excellent regeneration property. Referring to previously investigated influencing factors, pH and temperature had an evident influence on the removal of MB, and ionic strength had a finite effect. Therefore, a lower pH condition and temperature were adopted as the desorption condition. Here, the acidified water (pH 3) was adopted as the eluent, and the desorption process was performed at 303 K. The regenerated GO-COOH sponge has relative lower re-adsorption capability after lyophilization. The re-adsorption performance kept nearly constant even after five treatments, with a relative adsorption capacity for lyophilization treatment (23% of the initial capacity, Figure 7). The poor regeneration should be due to the strong electrostatic interaction between MB and GO-COOH sponge. According to the IR, the main signals were from the adsorbed MB molecules. The morphology had so few changes that the sheet structure became more distinguishable. Some sheets rolled under SEM. Nevertheless, MB molecules covered most adsorptive sites of GO-COOH and made recycling difficult for GO-COOH. In future studies, better regeneration protocols are needed.

## 4. Conclusions

In summary, GO-COOH sponge was prepared for the adsorption of MB, where the carboxylation of GO largely increased the adsorption capacity from 446 to 780 mg/g. The strong electrostatic interaction between carboxyl groups and MB molecules was believed to be the main reason for the increase in adsorption capacity. The adsorption behavior results collectively suggested that GO-COOH sponge was hopeful in treating cationic dyes and other positively charged pollutants. It is hoped that our results could benefit the development of graphene adsorbents and stimulate more interest in chemical modification of graphene adsorbents.

## Figures and Tables

**Table 1:** Linear fitting equations of different isothermal adsorption models.

Adsorption Model	Linear Fitting Equation	*R*
Langmuir	y = 0000216x + 0.00208	0.962
Freundlich	y = 0.144x + 5.706	0.979
Temkin	y = 73.26x + 263.75	0.956

**Table 2:** Adsorption capacities of graphene sponges for MB.

Adsorbent	*q_m_* (mg/g)	Ref.
GO sponge	446	[38]
GO-CS sponge	468	[39]
GO-Fe_3_O_4_ sponge	526	[40]
RGO sponge	27	[38]
RGO-MFe_2_O_4_	34.72	[41]
RL-GO	529.10	[42]
MCGO	95.16	[43]
GO-biopolymer gel	701.78	[44]
GO-COOH sponge	780 ± 47	This study

**Table 3:** Thermodynamics parameters of the adsorption of MB on GO-COOH sponge.

T (K)	*ΔG (kJ/mol)*	ΔH (kJ/mol)	ΔS (J/(mol·K))
303	−1.66	25.85	90.81
313	−2.57		
323	−3.48		
333	−4.39		
